# 47-year-old female with an apical mass

**DOI:** 10.1136/heartjnl-2016-310854

**Published:** 2016-12-21

**Authors:** Jack Andrews, Christopher CE Lang, Marc Dweck

**Affiliations:** 1 Centre for Cardiovascular Sciences, Chancellors Building, University of Edinburgh, Edinburgh, UK; 2 Edinburgh Heart Centre, Royal infirmary of Edinburgh, Edinburgh, UK

## Abstract

**Clinical introduction:**

A 47-year-old female with no medical history presented with a sudden collapse. Physical examination, chest X-ray and high-sensitivity cardiac troponin I were normal, however ECG demonstrated anterior T-wave inversion. CT pulmonary angiography was performed which ruled out pulmonary embolism but revealed a non-calcified, homogenous mass at the left ventricular (LV) apex. It was not clear whether this mass was intramyocardial or pericardial. Transthoracic echocardiography confirmed the apical mass but was unable to establish its aetiology. Subsequent cardiac MR (CMR) demonstrated a highly vascular intramyocardial mass on perfusion imaging (Figure 1A, online supplementary video A), with striking, homogenous late gadolinium enhancement (Figure 1B) consistent with a diagnosis of cardiac fibroma.^1^ The patient underwent successful surgical excision of the mass (see online supplementary image A) and made a good symptomatic recovery, quickly mobilising around the ward. On examination, the patient was afebrile but had a blood pressure of 90/40 mm Hg and raised venous pressure. Postoperative imaging with echocardiography (see online supplementary video B) and CMR (Figure 1C, D and online supplementary video C) revealed some unexpected findings. Study the provided images.

**Question:**

What is the next most appropriate management step?

- Antibiotic therapy for pericardial abscess
- Anticoagulation for LV thrombus
- Intravenous fluids with close clinical and imaging follow-up of the intramyocardial haemorrhage and pericardial haematoma
- Return to theatre for excision of residual tumour
- Urgent pericardiocentesis to drain pericardial collection

## ANSWER: C

**Figure 1:**
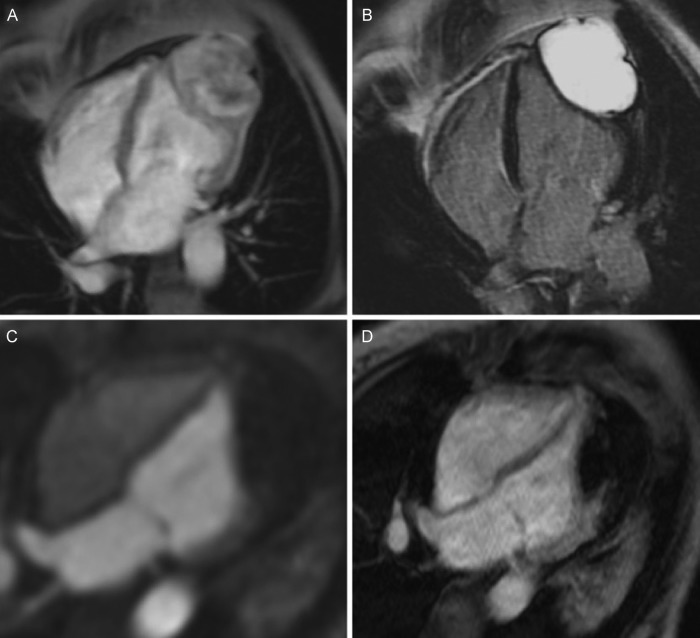
(A) Preoperative cardiac MR (CMR) perfusion. (B) Preoperative late gadolinium enhancement. (C) Postoperative CMR perfusion. (D) Postoperative early gadolinium enhancement.

Despite complete fibroma excision, postoperative echocardiography demonstrated a residual apical mass felt possibly to represent LV thrombus (see online supplementary video B). However, a repeat CMR again confirmed an intramyocardial apical mass (ruling out a mass or thrombus in the LV cavity) but with very different characteristics to preoperatively. First, it did not demonstrate a blood supply on perfusion imaging (Figure 1C, online supplementary video C) and second, it was associated with very low signal early after gadolinium administration (Figure 1D). These findings were therefore not consistent with residual tumour or pericardial abscess but instead indicated haemorrhage into the myocardial cavity previously occupied by the fibroma. Importantly, this haemorrhage also appeared to extend into the pericardial space, resulting in a large collection with a septal bounce and constrictive physiology now apparent on cine imaging (see online supplementary video D). In that context, both anticoagulation and pericardiocentesis were contraindicated in case further bleeding was precipitated. Discussion was held about the merits of repeat surgery and clot evacuation, however this was felt to be high risk with particular concern that postoperative bleeding at the site of this highly vascular tumour might again be encountered. A conservative management strategy was therefore adopted, with close echocardiographic follow-up demonstrating a reduction in the pericardial collection with time and reversal of the septal bounce (see online supplementary video E).

10.1136/heartjnl-2016-310854.supp1 supplementary figure

10.1136/heartjnl-2016-310854.supp2 supplementary video

10.1136/heartjnl-2016-310854.supp3 supplementary video

10.1136/heartjnl-2016-310854.supp4 supplementary video

10.1136/heartjnl-2016-310854.supp5 supplementary video

10.1136/heartjnl-2016-310854.supp6 supplementary video
