# Supplementary material for: 47-year-old female with an apical mass
Source: Heart. 2016 Dec 21;103(11):886. doi: 10.1136/heartjnl-2016-310854 (PMC5529987; doi:10.1136/heartjnl-2016-310854)

Supplementary Image A - Cranial view of the midline sternotomy with an open pericardium showing a 'pearly' irregular left ventricular mass protruding from the apex.

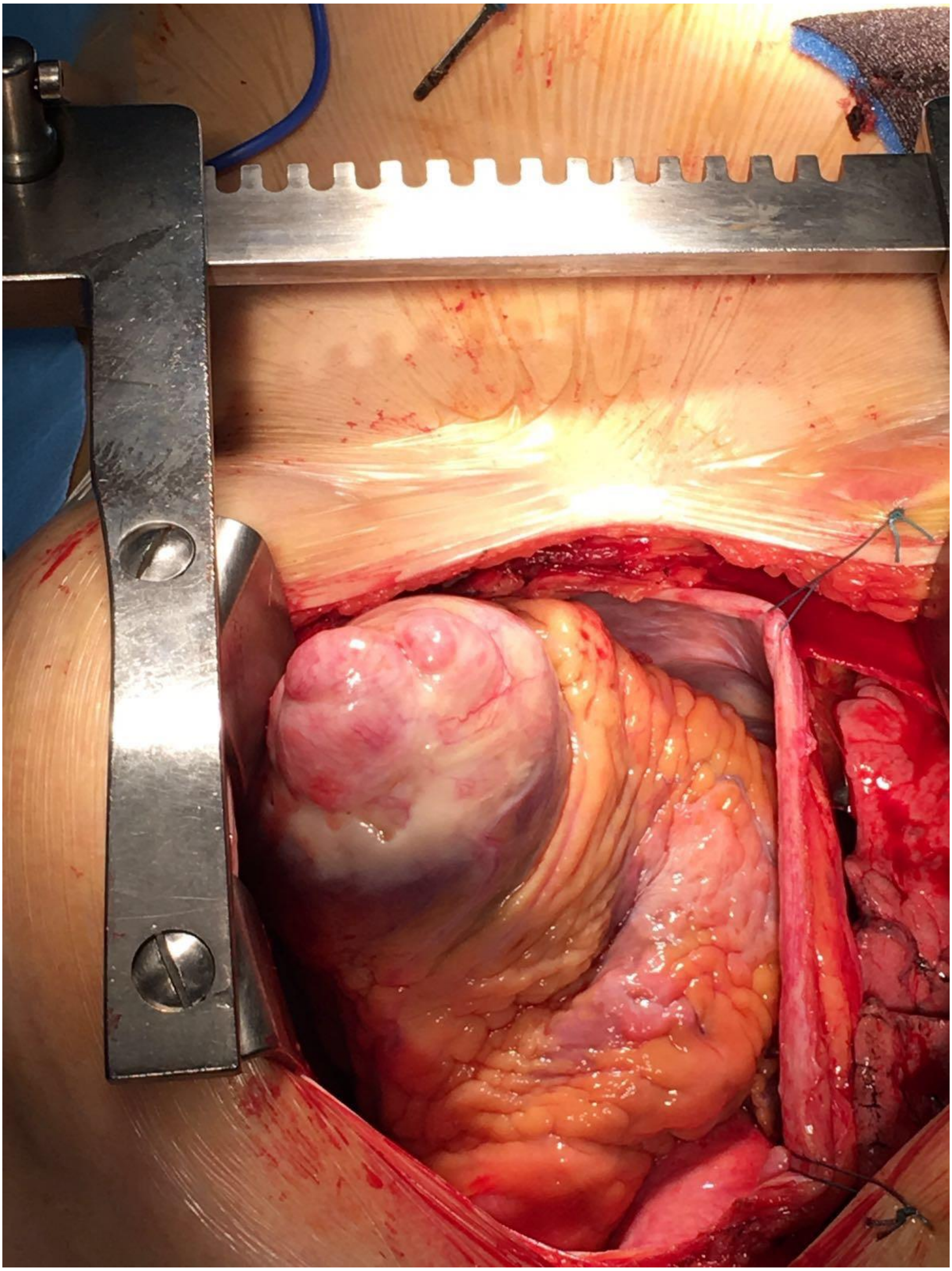

Supplement: supplementary figure [file heartjnl-2016-310854supp001.pdf]
